# Enhancing regeneration and repair of long-distance peripheral nerve defect injuries with continuous microcurrent electrical nerve stimulation

**DOI:** 10.3389/fnins.2024.1361590

**Published:** 2024-02-08

**Authors:** Junjie Kong, Cheng Teng, Fenglan Liu, Xuzhaoyu Wang, Yi Zhou, Ying Zong, Zixin Wan, Jun Qin, Bin Yu, Daguo Mi, Yaxian Wang

**Affiliations:** ^1^Key Laboratory of Neuroregeneration of Jiangsu and Ministry of Education, Affiliated Hospital and Medical School, Co-innovation Center of Neuroregeneration, Nantong University, Nantong, China; ^2^Department of Orthopedics, Nantong City Hospital of Traditional Chinese Medicine, Nantong, China

**Keywords:** peripheral nerve injury, long-distance nerve defects, Schwann cell activity, tissue engineering, functional recovery, continuous microcurrent electrical nerve stimulation

## Abstract

**Introduction:**

Peripheral nerve injuries, especially those involving long-distance deficits, pose significant challenges in clinical repair. This study explores the potential of continuous microcurrent electrical nerve stimulation (cMENS) as an adjunctive strategy to promote regeneration and repair in such cases.

**Methods:**

The study initially optimized cMENS parameters and assessed its impact on Schwann cell activity, neurotrophic factor secretion, and the nerve regeneration microenvironment. Subsequently, a rat sciatic nerve defect-bridge repair model was employed to evaluate the reparative effects of cMENS as an adjuvant treatment. Functional recovery was assessed through gait analysis, motor function tests, and nerve conduction assessments. Additionally, nerve regeneration and denervated muscle atrophy were observed through histological examination.

**Results:**

The study identified a 10-day regimen of 100uA microcurrent stimulation as optimal. Evaluation focused on Schwann cell activity and the microenvironment, revealing the positive impact of cMENS on maintaining denervated Schwann cell proliferation and enhancing neurotrophic factor secretion. In the rat model of sciatic nerve defect-bridge repair, cMENS demonstrated superior effects compared to control groups, promoting motor function recovery, nerve conduction, and sensory and motor neuron regeneration. Histological examinations revealed enhanced maturation of regenerated nerve fibers and reduced denervated muscle atrophy.

**Discussion:**

While cMENS shows promise as an adjuvant treatment for long-distance nerve defects, future research should explore extended stimulation durations and potential synergies with tissue engineering grafts to improve outcomes. This study contributes comprehensive evidence supporting the efficacy of cMENS in enhancing peripheral nerve regeneration.

## 1 Introduction

Peripheral nerve injuries represent a common clinical challenge, frequently arising from traumatic incidents or surgical procedures (Kornfeld et al., 2019; Lopes et al., 2022). For individuals facing long-distance nerve deficit injuries, where the gap between defects is so extensive that end-to-end suture techniques become intricate, the utilization of nerve grafts becomes an indispensable strategy (Wang et al., 2005). Traditionally, clinical repair relies upon autologous nerve grafting, widely regarded as the gold standard for nerve defect repair (Kornfeld et al., 2021). While this approach has shown effectiveness, it is not without its drawbacks, including the need for a secondary surgical site, limited donor nerve availability, and the potential for donor site morbidity (Gaudin et al., 2016).

In recent years, the field of regenerative medicine and tissue engineering has witnessed rapid developments. Tissue engineering grafts have gradually emerged as alternatives to traditional autologous nerve transplantation to repair peripheral nerve injuries (Zhang et al., 2022). However, despite these advancements, there remains a significant gap in the repair efficacy between tissue-engineered grafts and autologous nerves due to differences in structure and composition (Lischer et al., 2023). Nerve regeneration is inherently slow, with a rate of approximately 1 mm per day (Juckett et al., 2022), and the repair process can extend over months or even years, particularly in cases of long-distance or proximal nerve injuries. This prolonged denervation period often leads to severe consequences, such as muscle atrophy and joint contracture (Sawai et al., 2023), which can substantially affect the success of tissue-engineered grafts.

In response to the need for faster and more effective nerve regeneration strategies, many studies have explored the combination of cell transplantation (Yi et al., 2020), extracellular matrix support (Meder et al., 2021), neurotrophic factors (Li et al., 2020), and physical therapies, such as electrical stimulation (Chen et al., 2019; Chu et al., 2022), within the framework of tissue engineering repair. These studies have yielded promising therapeutic outcomes, emphasizing the potential of these approaches in enhancing peripheral nerve regeneration. Among these therapeutic modalities, electrical stimulation (ES) has a long history in the field of nerve injury repair, and its clinical efficacy has gained widespread recognition (ElAbd et al., 2022; Costello et al., 2023). Previous research has demonstrated that electrical stimulation can expedite the process of Wallerian degeneration (Li et al., 2023), encourage axonal regeneration (Zuo et al., 2020), and ultimately facilitate the restoration of motor and sensory functions.

The current clinical standard protocol involves brief intraoperative electrical stimulation lasting for 1 h at 20 Hz immediately after nerve repair (Ni et al., 2023). While achieving a balance between stimulation benefits and patient comfort, such brief, one-time stimulation may fall short for optimal or sustained results in long-distance injuries. Microcurrent electrical stimulation, characterized by current intensities typically below 1 milliampere (mA), operating in the low frequency and low-intensity spectrum, measured in microamperes (μA) (Zickri, 2014; Fujiya et al., 2015), provides minimal nerve stimulation (Poltawski et al., 2012; Hassan et al., 2020), ensuring patient comfort with high feasibility for continuous stimulation. Existing research has underscored the positive effects of microcurrent electrical stimulation on cellular physiology and regeneration (Kolimechkov et al., 2023). *In vitro* studies have demonstrated its ability to promote the directed migration of cells, including Schwann cells (Yao et al., 2015). Moreover, at the tissue level, microcurrents effectively reduce inflammatory cell infiltration (Lee et al., 2022), facilitating a rapid transition to the proliferative and remodeling phases of regeneration (Yu et al., 2014).

In light of these advances, we hypothesize that continuous microcurrent electrical nerve stimulation (cMENS) stands as a promising candidate for promoting nerve regeneration and facilitating functional recovery following long-distance peripheral nerve deficit injuries. This research endeavors to evaluate the potential of cMENS as an adjunctive strategy, comparing its efficacy to traditional autografts and assessing its application prospects in the field of peripheral nerve injury repair.

## 2 Materials and methods

### 2.1 Animals and surgical procedures

The animal experiments obtained ethical approval from the Administration Committee of Experimental Animals in Jiangsu Province, China (No. S20231120-003). Surgical procedures were conducted in accordance with the Institutional Animal Care Guidelines of Nantong University in Nantong, China. The entirety of the experiments was planned and documented in adherence to the guidelines specified by Animal Research: Reporting of *In Vivo* Experiments (ARRIVE).

**Figure 1:**
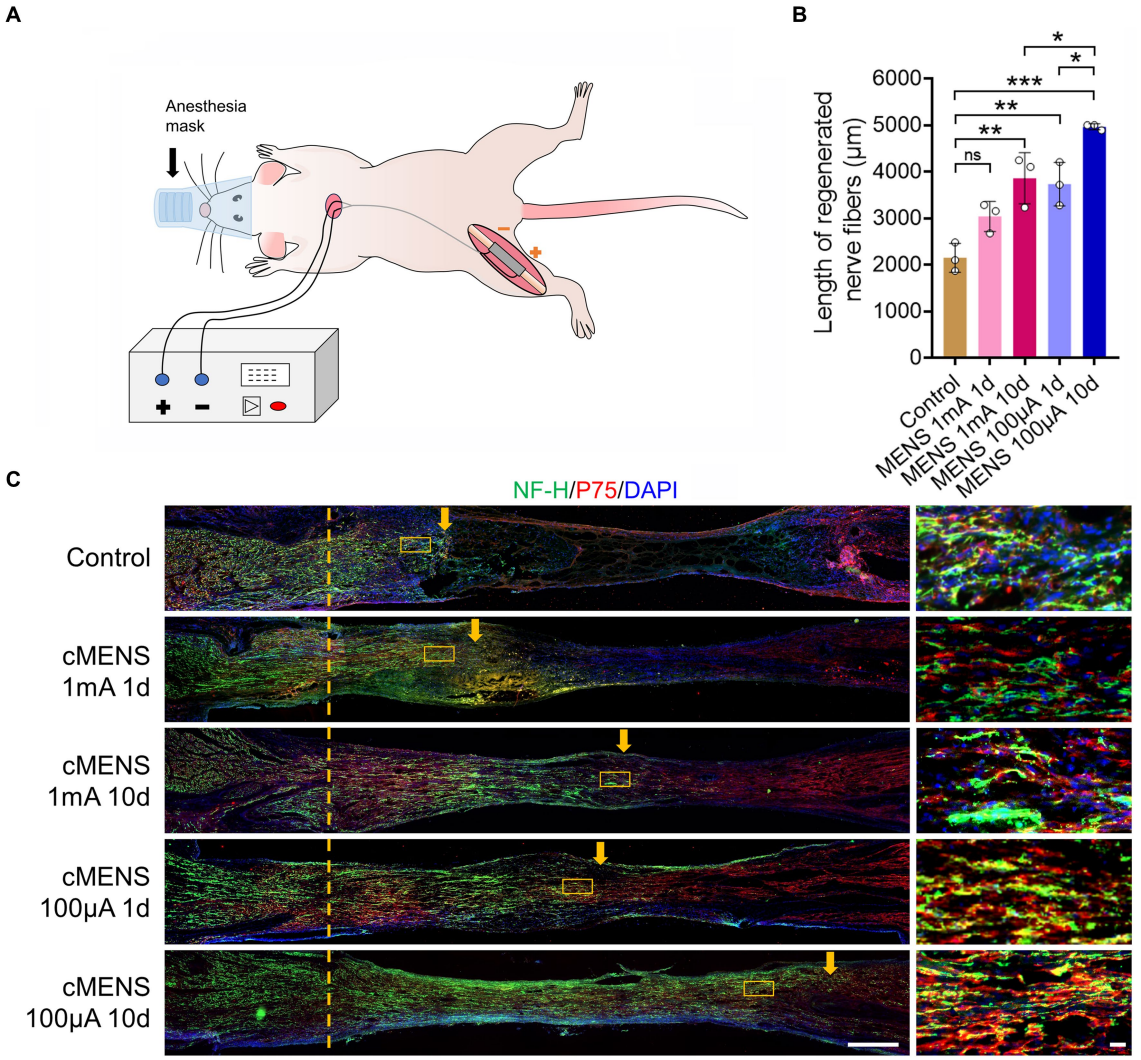
Effect of different electrical stimulation parameters on axon regeneration. **(A)** Schematic illustrating the experimental setup. A 5-mm sciatic nerve defect in rats was repaired using a silicone tube bridge. Flexible electrodes were attached to both tube ends, linked to the proximal and distal nerve stumps. These electrodes were threaded under the skin, securing their opposite ends to the dorsal neck skin. While receiving electrical stimulation, anesthetized rats were connected to a multi-channel stimulator via this electrode pair. **(B)** Representative immunofluorescence images depicting axon regeneration at the bridging site 10 days post-nerve repair. Arrows indicate the anterior part of the regenerating axon. The right images are magnification of the boxed areas in the left images. NF-H labels axons, P75 labels Schwann cells, and DAPI labels nuclei. Scale bar: left, 500 μm; right, 20 μm. **(C)** Quantitative analysis of the relative length of regenerated axons (*n* = 3). Data are presented as mean ± SEM and were statistically analyzed using one-way ANOVA, followed by Tukey *post hoc* test. *p < 0.05, **p < 0.01, ***p < 0.001, ns, not significant.

A total of 57 adult female Sprague–Dawley rats (2 months old, 180–220 g) were purchased from The Experimental Animal Center of Nantong University (license no. SCXK [Su] 2019-0001 and SYXK [Su] 2022-0046). Anesthesia was induced with isoflurane (1 L/min oxygen, 5% isoflurane) initially, and maintained with isoflurane (1 L/min oxygen, 1–3% isoflurane) during surgery (Ju et al., 2020). Subsequently, the left hind limb of the rats was shaved, sterilized, and an incision was made along the femur to expose the sciatic nerve. A segment of the sciatic nerve was excised and then reconstructed using a silicone tube (inner diameter: 12 mm, outer diameter: 20 mm, Chensheng, Shandong, China). The proximal and distal nerve stumps were individually sutured into the silicone tube using 8-0 sutures, creating a specified gap between the nerve stumps. To address the insulating properties of the silicone tube and ensure effective current conduction during microcurrent electrical nerve stimulation (MENS) application, the silicone lumen was filled with PBS buffer. A pair of flexible wire electrodes was then affixed to both ends of the silicone tube and connected to the proximal and distal nerve stumps. These electrodes were then subcutaneously threaded, with their opposite ends securely fastened to the dorsal neck skin. During electrical stimulation treatment, rats were anesthetized with isoflurane, and this electrode pair was linked to the positive and negative terminals of a multi-channel stimulator (BT-NSTIM BRAIN TECH, Jiangsu, China) (Figure 1A).

The control group underwent the same procedures, excluding the microcurrent stimulation. Additionally, two other experimental groups were established, namely the autologous nerve transplantation group and the defect group. In the former, a segment of the sciatic nerve was isolated, reversed, and subsequently re-sutured with the nerve stumps. In the latter, following the removal of a nerve segment, the proximal and distal nerve stumps were everted and sutured to adjacent muscle tissue. Finally, the muscle layers and skin were sutured, and post-operative care was administered to the rats until they fully recovered from anesthesia.

### 2.2 Real-time RT-PCR

At 4, 7, and 14 days following surgery, total RNA was extracted from the bridging nerve segment to serve as the template for RNA quantification, using Trizol isolation reagents (Invitrogen, Carlsbad, CA). RT-PCR was conducted on the StepOne real-time PCR system (Applied Biosystems, Foster City, CA, United States) with FastStart Universal SYBR Green Master (Roche, Basel, Switzerland). The primer sequences used were as follows:

bFGF (forward) 5′- ggagaagagcgacccaca-3′ and (reverse) 5′- gactccaggcgttcaaaga-3′.

BDNF (forward) 5′- caggggcatagacaaaag-3′ and (reverse) 5′- cttccccttttaatggtc-3′.

CNTF (forward) 5′- cctctgtagccgttctatctgg-3′ and (reverse) 5′- gtcgctctgcctcagtcatc-3′.

NGF (forward) 5′- gctggacccaagctcacc-3′ and (reverse) 5′-ccctctgggacattgctatc-3′.

GAPDH (forward) 5′- atgccatcactgccactca-3′ and (reverse) 5′- cctgcttcaccaccttcttg-3′.

The relative abundances of target genes were determined utilizing the 2^-ΔΔCt^ method.

### 2.3 Footprint analysis: sciatic functional index

The evaluation of motor function recovery occurred at 4, 8, and 12 weeks post-surgery using the CatWalk XT system (Noldus Information Technology, Wageningen, Netherlands), following established protocols (Chen et al., 2021). This system recorded paw movements and quantified 3D footprint intensities. The Sciatic Function Index (SFI) was computed using the following formula (Bain et al., 1989): SFI = −38.3[(EPL - NPL)/NPL] + 109.5[(ETS - NTS)/NTS] + 13.3[(EIT - NIT)/NIT] - 8.8.

In this equation, EPL represents the length of the experimental paw, NPL represents the length of the normal paw, ETS represents the experimental toe spread, NTS represents the normal toe spread, EIT represents the experimental intermediary toe spread, and NIT represents the normal intermediary toe spread. A SFI value of −100 indicates impaired nerve function, while a SFI value of 0 signifies normal nerve function.

### 2.4 Fine motor dynamic assessment

To conduct a comprehensive analysis of hind limb kinematics, fine motor function was assessed in distinct groups of rats 12 weeks post-injury employing the MotoRater system (TSE Systems, Germany). Each rat was positioned individually on a transparent track to evaluate their ground-walking performance. The TSE system enabled the precise identification of key anatomical landmarks, including the iliac crest, hip joint, knee joint, ankle and toe. This system facilitated the concurrent recording of three or more consecutive gait cycles. Finally, TSE Motion software was used to analyze the recorded videos and calculate the relevant parameters for each rat.

### 2.5 Compound muscle action potential recording

CMAP responses were assessed at 12 weeks following sciatic nerve injury, as previously described (Wang et al., 2015). In summary, rats were anesthetized deeply, and the designated region of the left hind limb was shaved. The sciatic nerve was re-exposed, and electrical stimuli were applied distal and proximal to the bridging segment alternately. CMAP responses in the left gastrocnemius muscle were recorded using the tendon–belly method (Korte et al., 2011). This involved inserting the active electrode into the mid-belly and the reference electrode into the achilles tendon of the gastrocnemius. Signal recording was conducted with the Keypoint 2 portable electromyograph (Dantec, Denmark). The recording process was repeated three times, and the amplitude and latency of the negative deflection were averaged for each rat. Motor nerve conduction velocity (MCV) was determined based on the distance and latency between the distal and proximal stimulated sites.

### 2.6 Fluorogold retrograde tracing

The fluorescent gold retrograde tracer experiment was performed 2 weeks before the end of the observation period. After deep anesthesia, the sciatic nerve on the injured side was re-exposed. Using a microsyringe, 3 μL 4% fluorogold solution (cat.no. 80014; Biotium, Fremont, CA, United States) was slowly injected into the epineurium in the distal nerve stump, approximately 5 mm beyond the bridge segment. The microsyringe was held in place for 2 min to prevent any liquid leakage, after which the incision was sutured. After a two-week interval, rats were fixed via perfusion with 4% paraformaldehyde, and the lumbar spinal cord, along with the corresponding dorsal root ganglia (DRG, L4-5) samples, were collected. Following dehydration, the tissues underwent frozen sectioning. Longitudinal sections of the spinal cord (25 μm thick) and dorsal root ganglion (12 μm thick) were examined under a fluorescence microscope (Axio Imager M2, Carl Zeiss Meditec AG, Jena, Germany), and cells exhibiting golden fluorescence were counted.

### 2.7 Immunohistochemical staining

Immunofluorescent staining was performed on sciatic nerve segments at various time points. The nerve tissues were fixed in 4% paraformaldehyde for 12 h. After gradual dehydration, the tissues were sectioned into 12-micrometer thickness using a cryostat microtome (Leica, Wetzlar, Germany). The prepared tissue sections were first treated with normal goat serum (Beyotime, Beijing, China) for 1 h at room temperature. Subsequently, the sections were incubated with primary antibodies, including chicken anti-NF-H (1:500; cat. no. ab134459; Abcam, Cambridge, MA, United States), rabbit anti-p75 (1:500; cat. no. ab52987; Abcam), and chicken anti-Ki67 (1:500; cat. no. NBP3-05538; Novus Biologicals, Littleton, CO, USA) at 4°C overnight. Then the sections were incubated with secondary antibodies: goat anti-chicken 488 (1:500; cat. no. ab150173; Abcam), goat anti-chicken 555 (1:500; cat. no. ab150174; Abcam), and goat anti-rabbit cy3 (1,500; cat. no. ab6939; Abcam) at room temperature for 2 h. The cell nuclei were counterstained with DAPI (Beyotime, Beijing, China). Finally, the sections were imaged using either a fluorescence microscope (Carl Zeiss) or a confocal microscope (Leica, Heidelberg, Germany).

### 2.8 Transmission electron microscopy

Transmission electron microscopy was utilized to examine nerve samples from the rats 12 weeks after the injury. Approximately 5 mm segments of the distal nerve stumps were promptly excised and subjected to the following procedures: they were quickly fixed in pre-chilled 4% glutaraldehyde, followed by post-fixation in a 1% osmium tetroxide solution. Subsequently, a stepwise dehydration process was carried out using progressively higher concentrations of ethanol, culminating in embedding the samples in Epon 812 epoxy resin. Ultrathin sections were stained with lead citrate and uranyl acetate, and they were then examined using a transmission electron microscope (HT 7700, HITACHI, Tokyo, Japan). The diameters of myelinated nerve fibers and their corresponding axons were measured, and myelin thickness and the G ratio were calculated using the following formulas: Myelin thickness = (myelinated fiber diameter - myelinated axon diameter)/2; G ratio = myelinated axon diameter/myelinated fiber diameter.

### 2.9 Muscle histological examination

At 12 weeks post-nerve injury, the gastrocnemius and tibialis anterior muscles on the left (injured) and right (uninjured) sides were collected and weighed. The wet weight of the left-side muscle was divided by that of the right-side muscle to determine the wet weight ratio. Subsequently, following a 12-h fixation at 4°C in 4% paraformaldehyde, the muscles were embedded in paraffin, and sliced into 6-μm thick transverse sections. Masson’s trichrome staining (catalog number G1340; Solarbio, Beijing, China) was applied to these tissue sections, coloring muscle fibers in red, collagen fibers in blue, and cell nuclei in black. The stained sections were then examined using an optical microscope (Carl Zeiss). The cross-sectional area (CSA) of muscle fibers and the area of proliferating collagen fibers were quantified using Image J software (Image J 1.50i, NIH, Bethesda, MD, United States).

### 2.10 Statistical analysis

Statistical analysis and graphing were performed using GraphPad Prism 8.0. Data is presented as mean ± SEM. Two-tailed Student’s t test, one-way ANOVA, and two-way ANOVA were used according to the experimental design. A *p*-value of less than 0.05 was considered statistically significant.

## 3 Results

### 3.1 Optimization of microcurrent electrical nerve stimulation parameters

To identify the optimal parameters of microcurrent electrical nerve stimulation (MENS), we conducted a screening process involving stimulus intensity and stimulus time. The following parameters were tested: (1) One-time stimulation, immediately after surgery, with a stimulation intensity of 1 mA (20 Hz, 200 us) for 15 min. (2) One-time stimulation, immediately after surgery, with a stimulation intensity of 100 uA (20 Hz, 200 us) for 15 min. (3) Continuous stimulation, with a stimulation intensity of 1 mA (20 Hz, 200 us), lasting for 15 min each session, administered once a day for 10 consecutive days. (4) Continuous stimulation, with a stimulation intensity of 100 uA (20 Hz, 200 us), lasting for 15 min each session, given once a day for 10 consecutive days.

Ten days after the initiation of these stimuli, rats were perfused, and the bridge segment of nerve tissue was collected to observe axon regeneration in animals from different groups. The results revealed that, in comparison to the control group, various parameters of electrical stimulation exhibited the potential to enhance axon regeneration (Figure 1B). Notably, the treatment involving a stimulus intensity of 100 uA delivered over 10 consecutive days yielded the most significant effect in terms of axon regeneration (Figure 1C). Consequently, it was determined that an intensity of 100 uA, applied for 15 min once a day over a 10-day period, represents the optimal parameters for the treatment of long-distance peripheral nerve defect injuries.

### 3.2 cMENS enhanced Schwann cell activity and microenvironment for nerve regeneration

**Figure 2:**
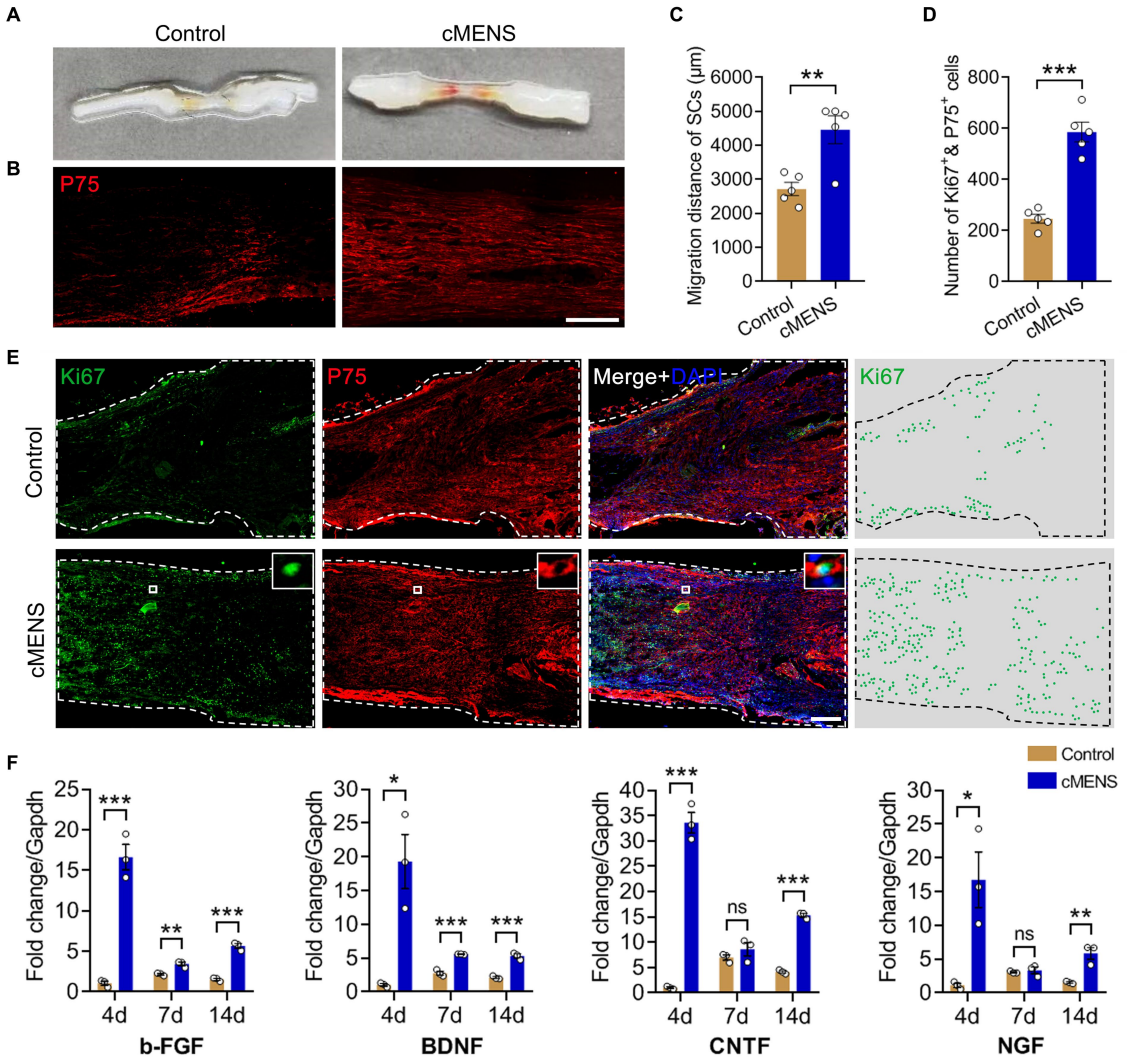
Effects of cMENS Treatment on the Proliferation and Migration of Schwann Cells (SCs) and Secretion of Neurotrophic Factors in the Local Microenvironment. **(A)** Gross observation of sciatic nerve at 10 days after 5 mm defection and bridging repair (the silicone tube has been removed). **(B)** Representative immunofluorescence image illustrating the migration of Schwann cells (SCs) in the bridging segment. P75 labels Schwann cells. Scale bar: 500 μm. **(C)** Quantification of SCs migrated distance (*n* = 5). **(D)** Quantification of proliferated SCs in the distal nerve stump (*n* = 5). **(E)** Representative immunofluorescence images depicting Schwann cell proliferation in the distal nerve stumps. Ki67 labels the proliferating cells, P75 labels Schwann cells, and DAPI labels nuclei. Scale bar: 200 μm. **(F)** Quantitative polymerase chain reaction (qPCR) analysis of neurotrophic factor secretion in the bridging segment. Data are presented as mean ± SEM and were statistically analyzed using Student’s *t* test (two-tailed, unpaired) **(C,D)**, and Multiple *t* tests **(F)**. *p < 0.05, **p < 0.01, ***p < 0.001, ns, not significant.

Prior studies have demonstrated that microcurrent electrical stimulation can promote the directed migration of Schwann cells *in vitro* (Yao et al., 2015). Here, we delve further into the impact of cMENS on Schwann cell proliferation, migration, and the local microenvironment. Ten days post-surgery, visual inspection revealed the formation of tissue connections between the proximal and distal nerve stumps in both the control group and the cMENS treatment group (Figure 2A). However, immunofluorescence results unveiled a significant disparity. In the control group, only a sparse population of migrating Schwann cells was observed within middle of the bridge segment, while the cMENS group exhibited an extensive assembly of migrating Schwann cells, forming a distinctive linear “regeneration channel” within the bridge segment (Figures 2B,C).

Immunofluorescence analysis of the distal nerve stumps 10 days after injury also revealed contrasting findings. In the control group, fewer Schwann cells proliferated within the distal nerve tissue, while the cMENS group displayed a substantial proliferation of Schwann cells in the distal nerve tissue (Figures 2D,E). Additionally, real-time quantitative PCR conducted at 4, 7, and 14 days post-surgery unveiled a notable increase in the secretion levels of various neurotrophic factors, including BDNF, NGF, CNTF, and bFGF, within the nerve tissue of the bridge segment in the cMENS group as compared to the control group (Figure 2F).

Collectively, cMENS treatment demonstrated the ability to sustain the proliferative activity of denervated Schwann cells, enhance their migration, elevate the release of various neurotrophic factors, and foster the creation of an optimal microenvironment for nerve regeneration.

### 3.3 cMENS facilitated functional recovery in long-distance peripheral nerve defect repair

**Figure 3:**
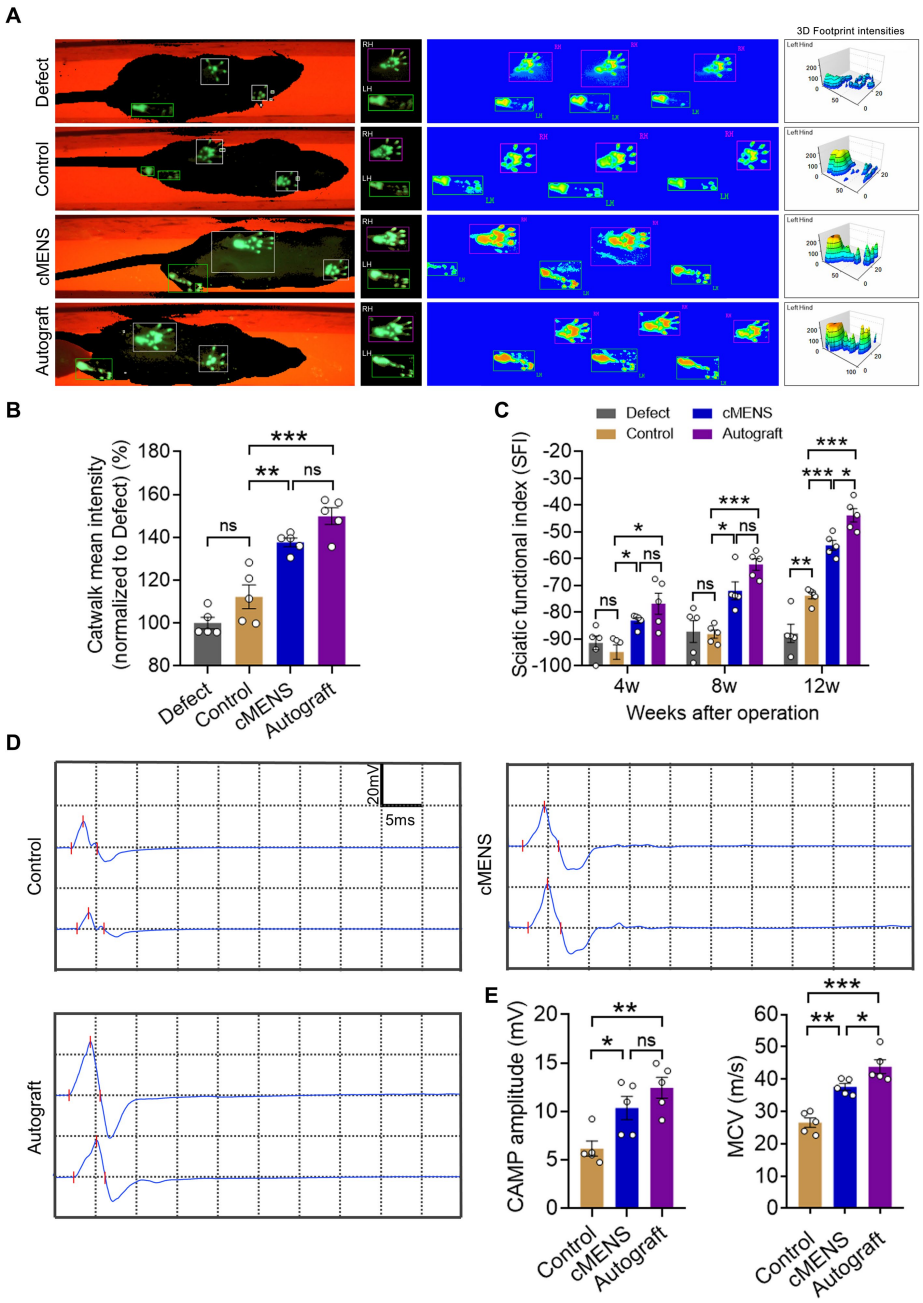
Effect of cMENS Treatment on the Improvement of Sciatic Nerve Function. **(A)** Representative footprint images 12 weeks after sciatic nerve 10 mm defect-bridging repair. LH represents the left hind paw (injured side), and RH represents the right hind paw (uninjured side). **(B)** Measurement of 3D footprint intensities 12w after nerve repair (*n* = 5). **(C)** Quantification of sciatic functional index (SFI) at 4, 8, and 12 weeks post-injury (*n* = 5). **(D)** Representative compound muscle action potential (CMAP) recordings at 12 weeks post-injury. **(E)** Quantification of peak amplitude of action potentials (*n* = 5). **(F)** Quantification of motor conduction velocity (MCV) (*n* = 5). Data are presented as mean ± SEM and were statistically analyzed using one-way ANOVA, followed by Tukey *post hoc* test **(B,E,F)**, and two-way ANOVA, followed by Tukey *post hoc* test **(C)**. *p < 0.05; **p < 0.01; ***p < 0.001; ns, not significant.

**Figure 4:**
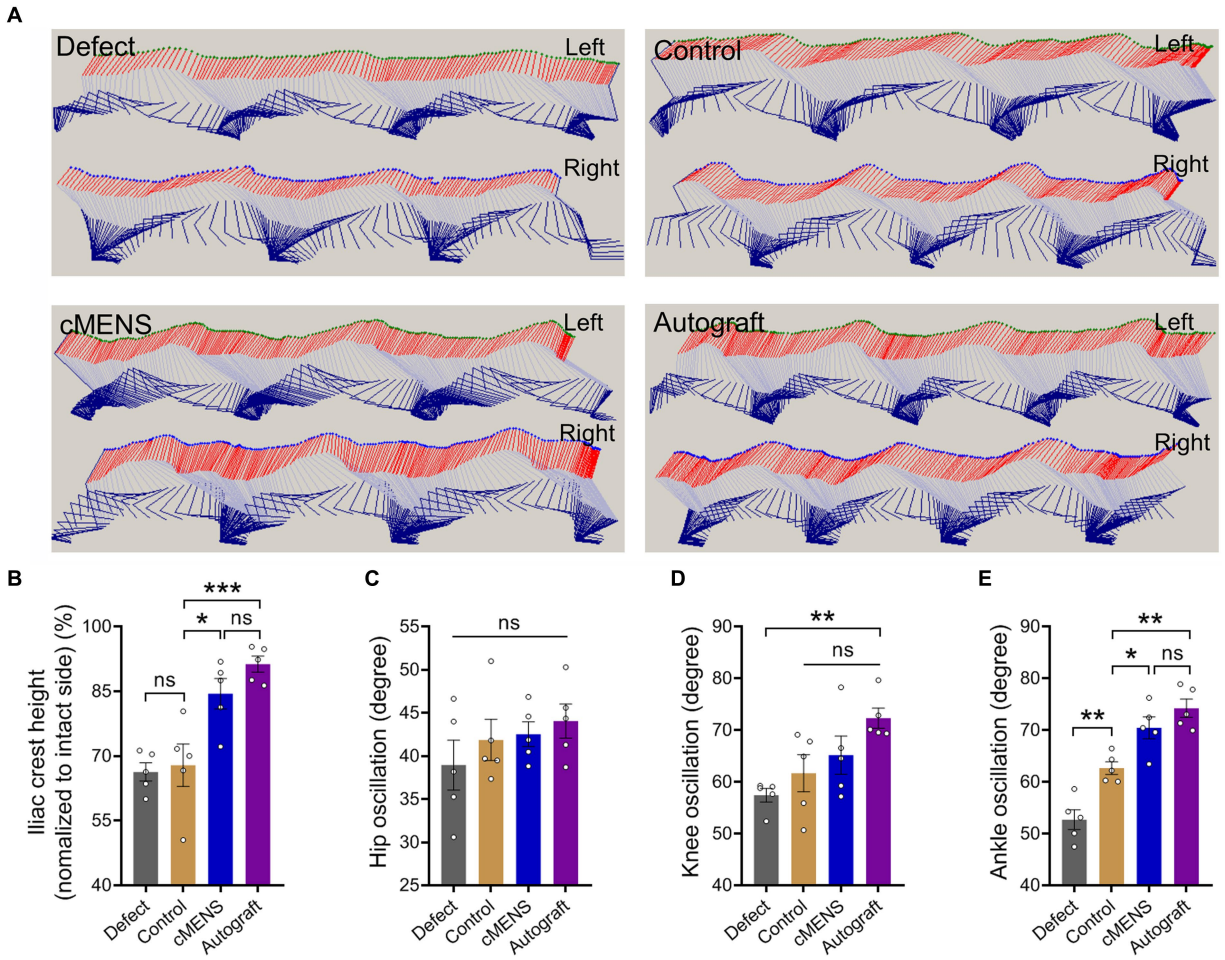
Effect of cMENS Treatment on Motor Function Recovery of the Injured Hindlimb. **(A)** Representative stick view decomposition of rat hindlimb movements during stance and swing at 12 weeks post sciatic nerve 10 mm defect-bridging repair. Left indicates the left hind paw (injured side), and Right indicates the right hind paw (uninjured side). **(B)** Quantification of iliac crest height (*n* = 5). **(C)** Quantification of hip joint swing angle during movement (*n* = 5). **(D)** Quantification of knee joint swing angle during movement (*n* = 5). **(E)** Quantification of ankle joint swing angle during movement (*n* = 5). Data are presented as mean ± SEM and were statistically analyzed using one-way ANOVA, followed by Tukey *post hoc* test **(B,C,D,E)**. *p < 0.05; **p < 0.01; ***p < 0.001; ns, not significant.

To evaluate the impact of cMENS treatment on limb motor function recovery following long-distance peripheral nerve deficit injury, we utilized the CatWalk gait analysis system at 4, 8, and 12 weeks post-operation. Comparative footprint analysis indicated varied degrees of motor function recovery in repaired groups compared to the non-repaired defect group (Figure 3A). At 12 weeks, the intensity measurement of the injured paw revealed significantly enhanced recovery in the cMENS group, approaching levels seen in the autologous group (Figure 3B). Periodic assessments of sciatic nerve function index (SFI) consistently underscored the substantial enhancement in motor function achieved by cMENS treatment, approaching the recovery levels seen in the autologous group (Figure 3C). In addition, using the TSE fine motor system, we captured hind limb movement trajectories during walking (Figure 4A). Kinetic analysis indicated minimal between-group differences in hip and knee joint oscillation (Figures 4C,D). However, the application of cMENS demonstrated significant enhancements in hind limb support height and ankle joint oscillation on the injured side, closely mirroring the recovery levels observed in the autologous group (Figures 4B,E).

To assess nerve conduction recovery, compound muscle action potential (CMAP) recordings were taken at 12 weeks post-operation (Figure 3D). Results showed significantly higher CMAP amplitudes in the cMENS and autologous groups compared to the control group, with no difference between the cMENS and autologous groups (Figure 3E). While the recovery of motor nerve conduction velocity (MCV) in the cMENS group outperformed the control group, a notable difference still existed compared to the autologous group (Figure 3F).

These findings suggest that cMENS treatment could be served as a facilitator for limb motor function and nerve conduction recovery in the context of long-distance peripheral nerve deficit injury.

### 3.4 cMENS promoted the nerve regeneration and maturation

**Figure 5:**
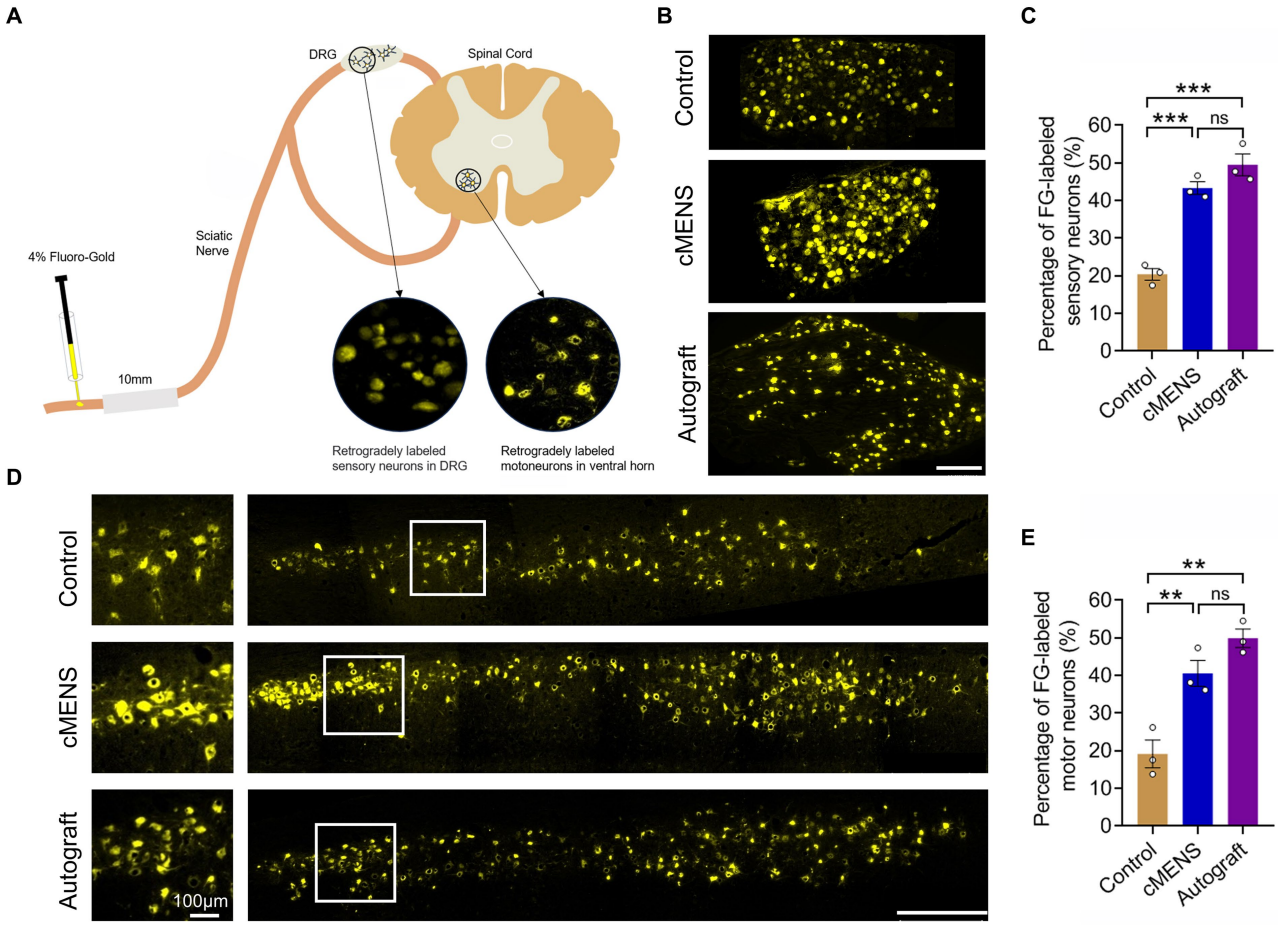
Effect of cMENS Treatment on the Regeneration of Sensory and Motor Neurons. **(A)** Schematic diagram of retrograde tracing of neurons. **(B)** Representative images displaying retrogradely labeled sensory neurons within the dorsal root ganglia (DRG) 12 weeks after nerve repair. Scale bar: 200 μm. **(C)** Quantification of FG-labeled sensory neurons (*n* = 3). **(D)** Representative images showing retrogradely labeled motor neurons within the ventral horn of the spinal cord 12 weeks after nerve repair. Magnified view of boxed areas displayed on the left side. Scale bar: left, 100 μm; right, 500 μm. **(E)** Quantification of FG-labeled motor neurons (*n* = 3). Data are presented as mean ± SEM and were statistically analyzed using one-way ANOVA, followed by Tukey *post hoc* test **(C,E)**. **p < 0.01; ***p < 0.001; ns, not significant.

Given the strong association between functional recovery and nerve regeneration, we next evaluated the regeneration status within each group. Twelve weeks post-operation, Fluorescence gold (FG) retrograde tracer detection indicated a substantial increase in the number of FG-labeled sensory neurons in the dorsal root ganglion (DRG) and FG-labeled motor neurons in the anterior horn of the spinal cord in the cMENS treatment group, compared to the control group. Remarkably, this increase approximated the numbers observed in the autologous nerve repair group, with no significant differences between the cMENS group and the autologous group (Figures 5A–E).

**Figure 6:**
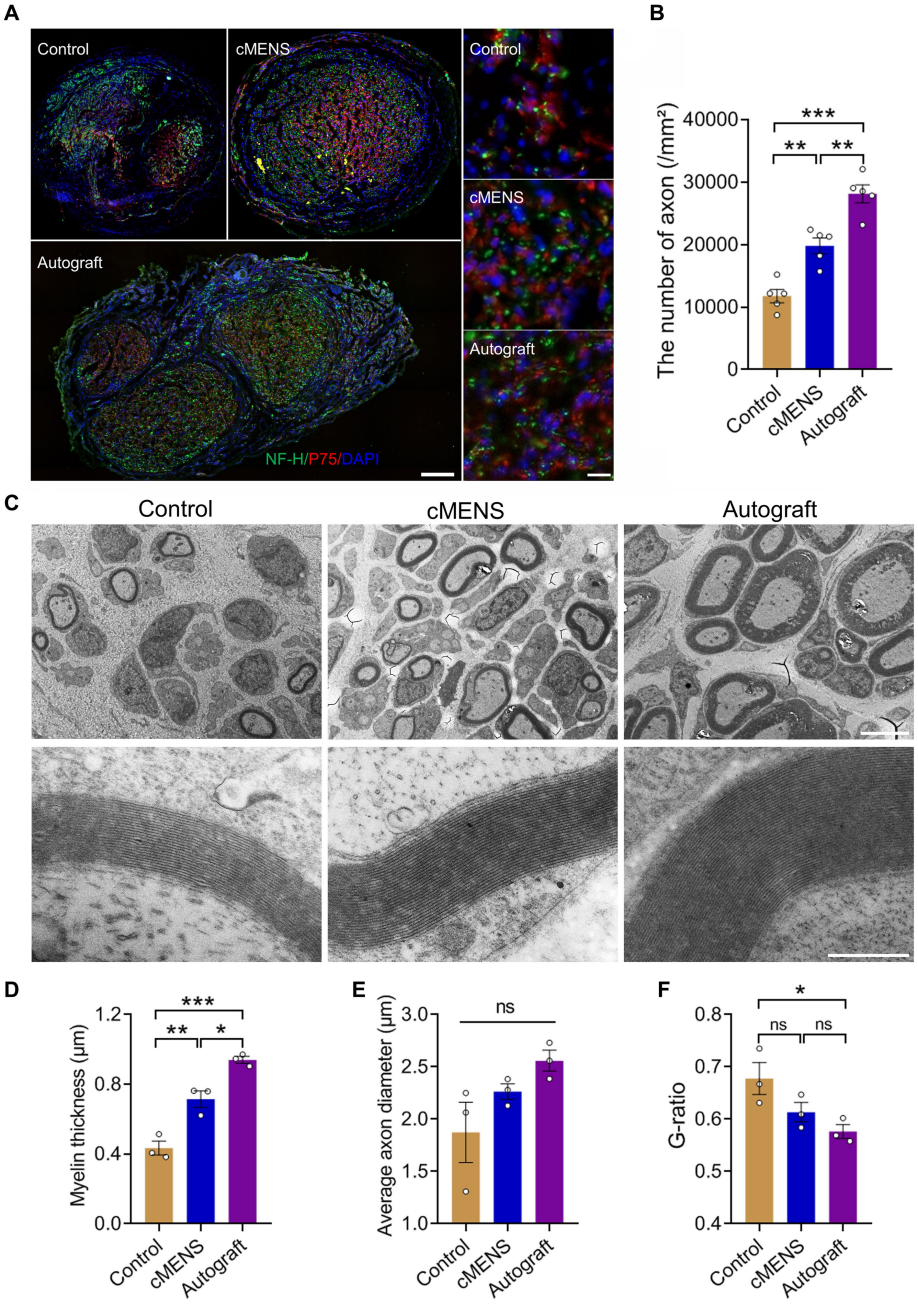
Effect of cMENS Treatment on Axon Regeneration and Myelination of Regenerating Axons. **(A)** Representative immunofluorescence images of transverse section of the distal part of the bridge segment at 12 weeks after sciatic nerve 10 mm defect-bridging repair. NF-H labels axons, P75 labels Schwann cells, and DAPI labels nuclei. Scale bar: left, 200 μm; right, 10 μm. **(B)** Quantification of the number of regenerated axons (*n* = 5). **(C)** Representative transmission electron microscopy images of myelinated nerve fibers at 12 weeks post-nerve repair. Scale bar: upper, 5 μm; lower, 500 nm. **(D)** Quantification of thickness of the myelin sheath (*n* = 3). **(E)** Quantification of axonal diameter of myelinated nerve fibers (*n* = 3). **(F)** G-ratio of myelinated nerve fibers (*n* = 3). Data are presented as mean ± SEM and were statistically analyzed using one-way ANOVA, followed by Tukey *post hoc* test **(B,D,E,F)**. *p < 0.05; **p < 0.01; ***p < 0.001; ns, not significant.

Immunofluorescence detection of distal nerve tissue in the bridge segment revealed varying degrees of nerve regeneration in each group following the defect. Notably, in comparison to the control group, both the cMENS treatment and autologous nerve repair group exhibited regenerated axons with a denser and more uniformly distributed pattern (Figure 6A). Despite improvement, the density of newly regenerated axons per unit area in the cMENS group remained slightly below that observed in the autologous group (Figure 6B).

Further investigation into remyelination of regenerated axons was conducted using transmission electron microscopy. The myelin sheath thickness of regenerated axons in the cMENS treatment group surpassed that of the control group significantly, though it still exhibited a noticeable difference compared to the autologous nerve repair group (Figures 6C,D). The autologous group demonstrated a clear advantage in terms of myelin thickness, axon diameter, and overall nerve maturation (Figures 6D–F).

These findings demonstrated that cMENS treatment not only promoted neuronal regeneration but also enhanced the maturation of regenerated nerve fibers.

### 3.5 cMENS alleviated denervated muscle atrophy

**Figure 7:**
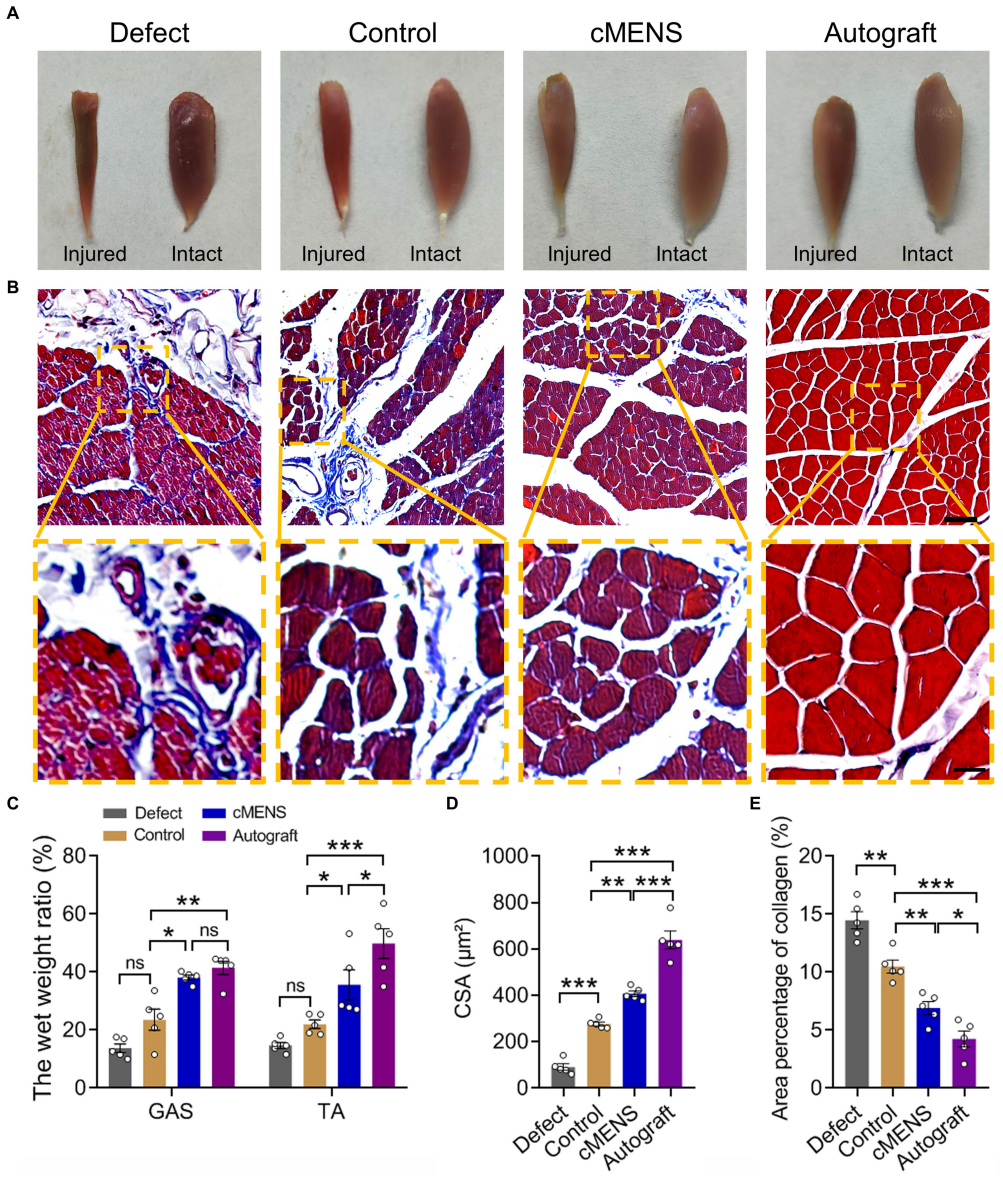
Effect of cMENS Treatment on Atrophy of Denervated Muscle. **(A)** Gross observation of tibialis anterior muscles on the injured and intact sides at 12 weeks after sciatic nerve 10 mm defect-bridging repair. **(B)** Representative images of Masson trichrome staining of the anterior tibial muscle on the injured side. Scale bar: upper, 50 μm; lower, 10 μm. **(C)** Wet weight ratio of the gastrocnemius (GAS) and tibialis anterior (TA) muscles (*n* = 5). **(D)** Quantification of the cross-sectional area (CSA) of muscle fibers in the tibialis anterior muscles (*n* = 5). **(E)** Average percentage of collagen fiber area in tibialis anterior muscle (*n* = 5). Data are presented as mean ± SEM and were statistically analyzed using two-way ANOVA, followed by Tukey *post hoc* test **(C)**, and one-way ANOVA, followed by Tukey *post hoc* test **(D,E)**. *p < 0.05; **p < 0.01; ***p < 0.001; ns, not significant.

Denervation-induced muscle atrophy is an inevitable consequence of long-distance peripheral nerve defects. Gross observations at 12 weeks post-operation revealed varying degrees of atrophy in the muscles on the injured side across all groups compared to their contralateral counterparts (Figure 7A). Analysis of muscle wet weight ratios, particularly for the gastrocnemius and tibialis anterior muscles, highlighted a significant recovery in the cMENS treatment group, closely approaching the levels seen in the autologous group. This recovery markedly surpassed that observed in the defect and control groups (Figure 7C).

Masson’s trichrome staining further demonstrated that, compared with the autologous nerve repair group, the other groups exhibited different degrees of collagen fiber proliferation. However, in contrast to the defect group and the control group, collagen fiber proliferation after cMENS treatment was significantly reduced (Figures 7B,E). Although muscle fiber atrophy was notably diminished after cMENS treatment, a discernible gap remained when compared to the outcomes achieved with autologous nerve repair (Figures 7B,D,E).

Together, these findings suggest that cMENS treatment effectively alleviated denervation-induced muscular atrophy, leading to significant improvements in muscle recovery.

## 4 Discussion

Recent decades have witnessed a surge in research efforts aimed at developing adjuvant therapies for enhancing peripheral nerve regeneration (Javeed et al., 2021). A well-established clinical practice involves the application of immediate electrical stimulation (ES) following nerve injury (Willand et al., 2015). This practice entails a one-time, brief intraoperative 1-h stimulation, which has gained preclinical and clinical support to promote early stages of regeneration, including neuronal survival and axonal sprouting (Calvey et al., 2015; Muzzi et al., 2019). However, regeneration after long-distance peripheral nerve deficit injuries is intricate and prolonged. Brief, one-time stimulation may prove insufficient for optimal or sustained results in such cases. Microcurrent electrical stimulation (MES) in the microcurrent (μA) range, similar to *in vivo* current, offers clinical benefits in tissue injury treatment and healing processes (Avendaño-Coy et al., 2022; Kolimechkov et al., 2023). Importantly, MES, as a subsensory stimulation modality (Poltawski et al., 2012; McMakin and Oschman, 2013), ensures patient comfort, making continuous stimulation highly feasible.

Before comprehensively evaluating the effect of continuous microcurrent electrical nerve stimulation (cMENS) on long-distance peripheral nerve defect repair, we optimized the electrical stimulation parameters. Given the established acceptance of a frequency of 20 Hz and pulse width of 200 us for peripheral nerve injury treatment (Park et al., 2019; Costello et al., 2023), our focus was on screening stimulation intensity and duration. Stimulation intensities of 1 mA and 100 uA, along with durations of one-time 15-min and continuous 10-day, 15-min sessions, were set in this study. In our rat sciatic nerve defect-bridge repair experiment, immediate ES significantly promoted axonal regeneration compared to the control group, consistent with current literature (Gordon, 2016). Notably, the effect of 100uA microcurrent stimulation was superior, with continuous 10-day microcurrent electrical nerve stimulation (cMENS) demonstrating the most significant impact. Thus, cMENS for 10 consecutive days emerged as the optimal parameter for treating long-distance peripheral nerve defect injuries.

Previous studies have elucidated that MES primarily exerts its effects at the cellular level by restoring cell membrane potential, enhancing electrical energy transport across cell membranes (Kang et al., 2015; Hassan et al., 2020). Building on these positive cellular effects, we assessed the impact of cMENS on Schwann cells (SCs), the key supporting cells in peripheral nerve regeneration (Jessen and Mirsky, 2019; Rao et al., 2022). Immunohistochemical staining revealed a higher density of SCs in the bridging segment tissue of the cMENS group on day 10 after surgery. Migrated SCs formed a continuous “regeneration channel,” connecting the proximal and distal nerve tissue. Furthermore, cMENS significantly enhanced the proliferation of SCs in the distal stump and increased the secretion of neurotrophic factors, affirming its positive role in maintaining SC proliferation and improving the regeneration microenvironment.

With the positive role of cMENS in nerve injury repair established, we further evaluated its effectiveness as an adjuvant treatment for rat sciatic nerve long-distance defect injuries. Footprinting tests, TSE fine motor analysis, and nerve conduction detection collectively demonstrated that cMENS significantly promoted the recovery of motor function in the injured limb compared to the control group. The treatment also enhanced the limb’s ability to support body weight, improved ankle joint swing angles during walking, and facilitated the recovery of nerve conduction function in regenerated nerve fibers. Histological examinations further supported the positive impact of cMENS on sensory and motor neuron regeneration, as well as the maturation of regenerated nerve fibers. Additionally, cMENS significantly reduced the degree of atrophy in denervated muscles.

While the potential of cMENS as an adjuvant treatment for long-distance peripheral nerve defect repair has been confirmed, and limb motor function recovery is close to that of autologous nerve transplantation repair, significant disparities remain in nerve conduction, maturation of regenerated nerve fibers, and reduction of denervated muscle atrophy. We speculate that these disparities may be attributed to the relatively short duration of continuous stimulation (10 days) and the use of silicone tube bridge repair in this study. The inherent advantages of autologous nerve repair, such as the natural composition of cell components, tissue structure, and the presence of rich nutritional factors, contribute to the superior performance of autologous nerve repair compared to the silicone tube bridging repair used in our study. To address or narrow this gap in future studies, we propose several strategies. Firstly, considering the limitations of the silicone tube, we are exploring more advanced tissue engineering grafts with a finer structure, incorporating cytokines, and utilizing extracellular matrix to better mimic the natural nerve environment. This approach may enhance the regenerative potential of the repair strategy. Secondly, regarding the duration of continuous electrical stimulation, which was limited to 10 days in our study due to electrode fragility, we are actively investigating electrodes with a longer service life. Prolonging the stimulation period beyond 10 days could potentially lead to further improvements in the efficacy of electrical stimulation.

In conclusion, our study provides comprehensive evidence supporting the efficacy of continuous microcurrent electrical nerve stimulation (cMENS) in enhancing the regeneration and repair of long-distance peripheral nerve defects. Meanwhile, it is essential to acknowledge current limitations, such as electrode implantation invasiveness and observed disparities compared to autologous nerve repair, underscores the need for ongoing research and development. Future studies should explore less invasive cMENS delivery methods, such as bioresorbable implantable wireless nerve stimulators (Koo et al., 2018), and investigate the synergistic potential of cMENS with current tissue engineering nerve grafts to ensure its viability as an option for assisting in the repair of long-distance peripheral nerve defects.

## Data availability statement

The original contributions presented in the study are included in the article/supplementary material, further inquiries can be directed to the corresponding authors.

## Ethics statement

The animal study was approved by the Administration Committee of Experimental Animals in Jiangsu Province, China. The study was conducted in accordance with the local legislation and institutional requirements.

## Author contributions

JK: Writing – original draft, Software, Data curation, Investigation, Methodology. CT: Data curation, Writing – original draft, Investigation. FL: Methodology, Writing – original draft, Investigation. XW: Writing – original draft, Investigation. YZh: Writing – original draft, Investigation. YZo: Writing – original draft, Investigation. ZW: Writing – original draft, Investigation. JQ: Writing – review & editing, Conceptualization. BY: Writing – review & editing, Conceptualization. DM: Writing – review & editing, Conceptualization, Supervision, Validation. YW: Writing – review & editing, Writing – original draft, Conceptualization, Funding acquisition, Project administration, Supervision, Validation.
